# Changes in Vitamin D Status in Overweight Middle-Aged Adults with or without Impaired Glucose Metabolism in Two Consecutive Nordic Summers

**DOI:** 10.1155/2019/1840374

**Published:** 2019-03-03

**Authors:** Petra Lundström, Kenneth Caidahl, Maria J. Eriksson, Tomas Fritz, Anna Krook, Juleen R. Zierath, Anette Rickenlund

**Affiliations:** ^1^Department of Molecular Medicine and Surgery, Karolinska Institutet, Stockholm, Sweden; ^2^Department of Clinical Physiology, Karolinska University Hospital, Stockholm, Sweden; ^3^Department Department of Molecular and Clinical Medicine, Institute of Medicine, Sahlgrenska Academy, University of Gothenburg, Gothenburg, Sweden; ^4^Department of Physiology and Pharmacology, Karolinska Institutet, Stockholm, Sweden; ^5^Novo Nordisk Foundation Center for Basic Metabolic Research, University of Copenhagen, Copenhagen, Denmark

## Abstract

**Background:**

Sun exposure is the main driver of vitamin D synthesis. High latitude, obesity, and type 2 diabetes mellitus (T2DM) are all risk factors for vitamin D deficiency. However, the seasonal variation in vitamin D concentrations (25[OH]D) in such populations before and after sun exposure during the summer is unknown. Therefore, we investigated 25[OH]D status before and after two consecutive summers in high latitude and its associations with body fat, sex, and glucose metabolism.

**Methods:**

158 participants from Sweden (87 women, 71 men; mean age, 60 ± 5 y; body mass index ≥ 25 kg/m^2^) and 25[OH]D were measured and evaluated in relation to normal or impaired glucose tolerance, body composition, and dietary habits during summer season.

**Results:**

Eighty-four percent of the participants were categorized with low to deficient 25[OH]D values before summer (55.1 ± 21.7 nmol·L^−1^), with a significant increase after the summer season (66.3 ± 21.0 nmol·L^−1^; *P* < 0.001). However, the values remained below the recommended range (76–250 nmol·L^−1^) in 66% of the participants. These findings were verified in a subgroup of the study population during the subsequent summer. Participants who reported use of vitamin D supplements had higher initial concentrations (64.2 ± 20.1 nmol·L^−1^) compared to nonusers (53.7 ± 21.7 nmol·L^−1^; *P*=0.04). Further, 25[OH]D values correlated negatively with fat mass (kg) prior to summer only in the female population (*r*=−0.29,  *P*=0.008).

**Conclusions:**

In the present study, sun exposure had a beneficial but insufficient effect on 25[OH]D levels, and the same levels were documented in two consecutive summer seasons, confirming that vitamin D supplementation in both summer and winter should be considered in this population.

## 1. Introduction

The vitamin D endocrine system is linked with obesity and type 2 diabetes mellitus (T2DM) [1, 2], and there has been an increased interest regarding associations between vitamin D, measured as serum 25-hydroxyvitamin D 25[OH]D, body mass index (BMI), and total body fat. A reasonable explanation may be that high total fat mass cause lower bioavailability of vitamin D [3]. Further, a low serum concentration of 25[OH]D has shown to correlate with glucose intolerance, insulin resistance, and T2DM, which are all implicated in the metabolic syndrome [4, 5]. Suggested mechanisms for the positive action of vitamin D in insulin physiology are enhancement of *β*-cell function in the pancreas and improved insulin sensitivity in target cells (liver, skeletal muscle, and adipose tissue) [6].

Cholecalciferol, also known as vitamin D_3_, is primarily synthesized via ultraviolet B (UVB) irradiation of the dermis, where it is converted to its biologically active form, 1,25-dihydroxyvitamin D, through hydroxylation in the liver and kidney [7]. Further, vitamin D is found in small quantities in supplements and in foods such as fatty fish and fortified milk [8].

Low concentrations of 25[OH]D are mainly attributable to limited sunlight exposure caused by low UVB exposure at high latitudes, an indoor lifestyle, air pollution, sunscreen, and clothing [9, 10]. Confounding factors include poor gastrointestinal absorption of vitamin D, sex, and age [8].

The aim of this study was to investigate the effects of sun exposure by assessing vitamin D levels prior to and after two consecutive northern latitude summer in a middle-aged overweight population with either normal or impaired glucose metabolism.

A second aim was to study the associations between 25[OH]D, body composition, vitamin D supplementation, and intake of fatty fish.

## 2. Participants and Methods

### 2.1. Participants

**Figure 1:**
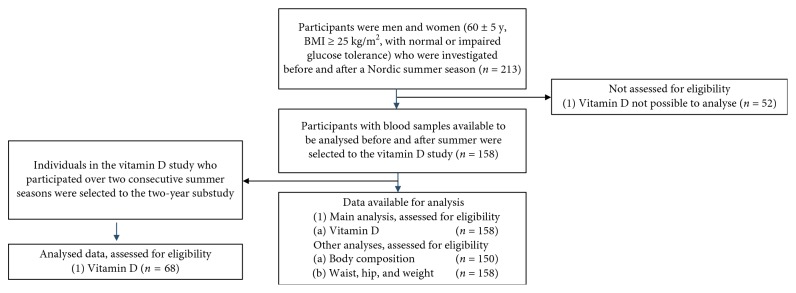
The flow chart of recruitment procedures.

Participants were selected from individuals enrolled in a larger randomized control study, whereas vitamin D values were analysed prior to and after the summer season. The recruitment and randomization process is described briefly in Figure 1. For further details, see Fritz et al 2013 [11]. The 158 participants were Caucasian (age range, 45–69 y) with a BMI ≥ 25 kg/m^2^ and were living in the Stockholm area, Sweden, at 59°N latitude.

An oral glucose tolerance test (OGTT) was performed at the time of inclusion into the study. Participants were classified into two groups: normal glucose tolerance (NGT; <8.9 mmol/L) and impaired glucose tolerance (IGT 8.9–12.1 mmol/L)/T2DM (≥12.2 mmol/L) according to their 2 h glucose concentration after OGTT. Insulin treatment was used as an exclusion criterion as it interferes with the calculation of insulin resistance [11]. Written informed consent was obtained from all participants, and the study protocol was approved by the Ethics Committee of Karolinska Institutet, Stockholm.

### 2.2. Biochemical Methods

All blood samples used for biochemical tests were frozen at −70°C and analysed at the Karolinska University Hospital Laboratory. The concentration of plasma glucose was determined in a fasting state, prior to the ingestion of 75 g of glucose in water, repeated after 2 h. The mean concentrations in two capillary blood samples were calculated by using the homeostasis model assessment—insulin resistance model, and insulin resistance was calculated as fasting insulin (mU/mL) × fasting glucose (mmol/L)/22.5. Further, 25[OH]D was determined using the Liaison 25 OH Vitamin D assay (DiaSorin, Saluggia, Italy) at the Karolinska University Hospital Laboratory. This method is a direct competitive chemiluminescence immunoassay for the quantitative determination of total 25[OH]D in serum or plasma on an automated platform [12]. The DiaSorin analysis has a range of detectable 25[OH]D from 10.0 nmol·L^−1^ to 375 nmol·L^−1^, with a good concordance correlation coefficient of 0.978 [13]. We applied the following definitions of 25[OH]D status [14, 15]: deficient (<30 nmol·L^−1^), insufficient (30–50 nmol·L^−1^), low (51–75 nmol·L^−1^), and adequate (76–250 nmol·L^−1^).

### 2.3. Body Composition Measurements

Body composition was determined using Bod Pod software (version 1.68; Life Measurement Inc., Concord, CA, USA). Bod Pod is a noninvasive dual-chamber plethysmograph that utilizes a densitometry method using air displacement [16]. Bod Pod software is applied to divide the body into a two-component model: fat mass (FM) and fat-free mass using an equation derived originally by Siri [17]. The day-to-day variation in our laboratory was measured over 5 consecutive days 1 week before the study, in one male and two females. The coefficient of variation for FM was estimated to be 2% in accordance with Fields et al. [18]. Body weight (wt) was determined on a calibrated scale connected to the Bod Pod. The participants' waistline and hips were measured according to the International Standardization of Anthropometry and Kinanthropometry. Participants were instructed to avoid strenuous physical activity for 24 h and to report to the laboratory after a 4 h fast prior to the measurements.

### 2.4. Self-Reported Dietary Supplementation and Fatty Fish Intake

All 158 participants completed a questionnaire regarding dietary habits and supplementation that included questions about their intake of fatty fish. They ranked the frequency of fatty fish intake in the following order: once per month, once per week, 1–3 times per week, and 3 times or more per week. The questionnaire was completed at baseline and after the first summer. The food questionnaire was based on the recommendations from National Food Agency, Sweden.

### 2.5. Statistical Analyses

25[OH]D levels before and after summer in the different groups within the study population are presented as mean values and standard deviations (SDs). Any association between 25[OH]D and other variables was assessed by computing Pearson's correlation coefficients.

To investigate the determinants of 25[OH]D levels, the study population was first analysed as a whole group (158). Next, women and men were analysed separately and then further allocated to groups according to glucose metabolic capacity. 25[OH]D levels before and after the summer season, or seasonal changes in 25[OH]D levels, were used as the dependent variable in multiple linear regression analyses. The explanatory value (*R*^2^) for 25[OH]D levels was analysed in a multivariable model including fat (kg or %), age, sex, glucose metabolism status, fish intake, and vitamin D supplementation. Statistical analyses were performed using IBM SPSS Statistics for Windows, version 25.0 (IBM, Chicago, IL, USA).

## 3. Results

### 3.1. Seasonal Effects on Serum Concentrations of 25[OH]D

A total of 158 participants (87 women, 71 men; mean age, 60 ± 5 y) participated in the study. The results for 25[OH]D levels before and after summer for four different groups based on glucose metabolism status and sex are presented in Table 1. We repeated the measurements in a subpopulation of 68 participants the following summer. No significant differences were found in the mean values of 25[OH]D before or after the second summer compared with the first year (Table 1).

**Figure 2:**
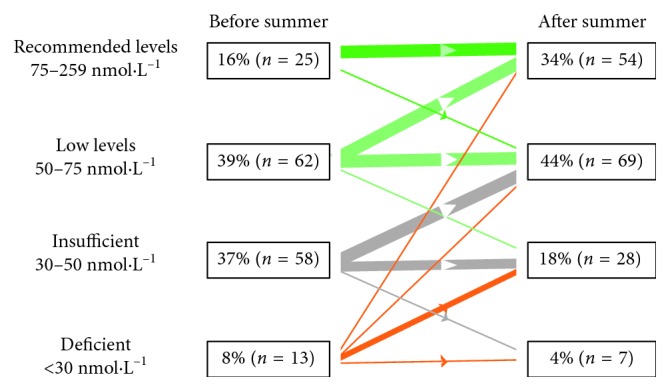
The distribution of the study participants according to their 25[OH]D concentrations before and after summer, grouped into four categories: deficient, insufficient, low, and adequate.

The mean concentration of 25[OH]D for the whole study population prior to summer was in the low to deficient range (55.1 ± 21.7 nmol·L^−1^) (Figure 2). After the 4-month summer period, the concentration had increased to 66.3 ± 21.0 nmol·L^−1^ (*P* < 0.001); however, it was still below the recommended range in the majority of participants (Figure 2).

Figure 2 shows the distribution of the study participants according to their 25[OH]D concentrations grouped into four categories: deficient, insufficient, low, and adequate.

### 3.2. Vitamin D Supplementation and 25[OH]D Concentrations

The vitamin D dietary supplements reported by the participants included different products with a mean vitamin D content of 7.5 *μ*g; however, only 21 participants (13% of the study population) reported taking supplements. The mean 25[OH]D concentration among supplement users was higher prior to summer (64.2 ± 20.1 nmol·L^−1^) compared with that of nonusers (53.7 ± 21.7 nmol·L^−1^; *P*=0.04). After summer, the concentrations in supplement users were 71.1 ± 22.8 nmol·L^−1^ compared with that of nonusers (65.6 ± 20.7 nmol·L^−1^). The seasonal change in 25[OH]D was significant for both groups, supplement-users (*P*=0.003), and nonusers (*P* < 0.001).

### 3.3. Correlations with Body Composition Variables and 25[OH]D Concentrations

Correlation analysis with 25[OH]D as the dependent variable for participants whose body composition was measured (*n*=150) demonstrated an inverse significant correlation between 25[OH]D and body composition variables before summer (Table 2). Due to sex differences for certain body composition variables, separate correlation analyses were performed for women and men. We found inverse correlations prior to summer between 25[OH]D and the following body composition variables in women (*n*=82): BMI (*r*=−0.28,  *P*=0.009), FM (%, *r*=−0.23, *P*=0.04; kg, *r*=−0.29, *P*=0.008); wt (*r*=−0.29, *P*=0.008) and hip circumference (*r*=−0.35,  *P*=0.001). Waist was not significant in women before summer. After summer, the degree of correlation between vitamin D and body composition variables at baseline remained essentially unchanged: BMI (*r*=−0.34,  *P*=0.001), FM (%, *r*=−0.38, *P*=0.001; kg, *r*=−0.39, *P* < 0.001), wt (*r*=−0.34,  *P*=0.002), waist (*r*=−0.27,  *P*=0.01), and hip circumference (*r*=−0.38,  *P* < 0.001). In men (*n*=69), no correlation was found between 25[OH]D and body composition variables.

**Figure 3:**
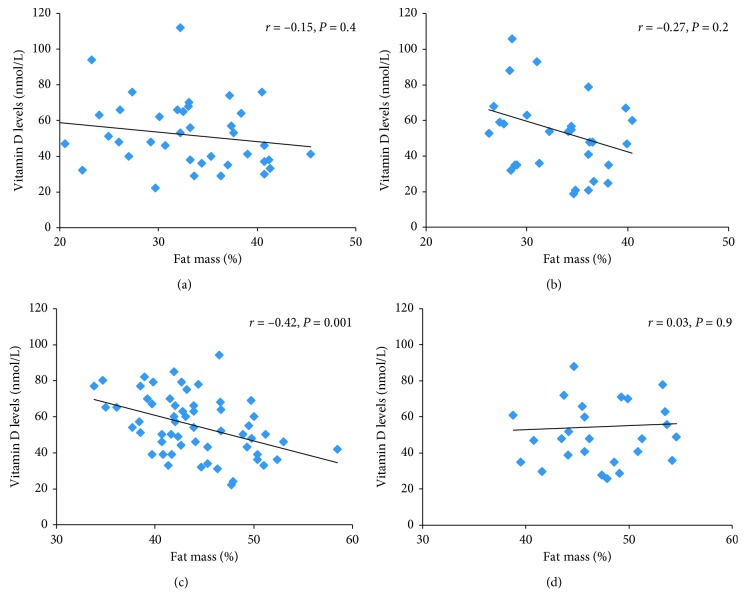
Pearson's correlations between levels of 25[OH]D and fat mass (%) in four groups according to gender and glucose metabolic state; NGT Men = men with normal glucose tolerance, IGT + T2DM Men = men with insulin resistance and diabetes mellitus type 2. NGT Women = women normal glucose tolerance, IGT + T2DM Women = women with insulin resistance and Type 2 diabetes mellitus. *R* value and *p* value are indicated in the graphs.

When we allocated participants into groups according to both sex and glucose metabolism, we found weak negative correlations at baseline between 25[OH]D and body composition in women with NGT (*n*=57), BMI (*r*=−0.28,  *P*=0.03) FM related variables (%, *r*=−0.42, kg, *r*=−0.44, both *P*=0.001), wt (*r*=−0.36,  *P*=0.006), and waist and hip circumference (*r*=−0.38,  *P*=0.03 and *r*=−0.44,  *P*=0.001, respectively). The group correlations with FM (%) prior to summer are shown in Figure 3. No correlation was found between the frequency of self-reported fatty fish intake and 25[OH]D (Table 2). After summer, the correlations between vitamin D and body composition variables increased to moderate in women with NGT : BMI (*r*=−0.37,  *P*=0.003), FM (%, *r*=−0.47,  *P* < 0.001; kg, *r*=−0.51,  *P* < 0.001), wt (*r*=−0.44,  *P*=0.001), waist (*r*=−0.37,  *P*=0.005), and hip circumference (*r*=−0.49,  *P* < 0.001).

After summer, the correlations between vitamin D and some body composition variables in women with NGT further increased.

In a multiple regression model for the whole study population, we included the significant variables from univariate analyses at baseline: vitamin D supplementation and one of the variables expressing body composition (FM, kg), together with nonsignificant variables: age, sex, fish intake, and glucose metabolism, all of which are potential determinants of 25[OH]D. We found that the overall *R*^2^ for the model was weak but significant (Table 3). Further, we divided the study population into groups according to sex and analysed the effect size for FM (%). A slightly stronger overall *R*^2^ was observed for the model in women than for the whole study population (Table 4). In summary, the level of explanation of contributing factors to 25[OH]D was low.

## 4. Discussion

To our knowledge, this is the first study to investigate 25[OH]D levels before and after two consecutive summer seasons in an overweight population with and without impaired glucose metabolism. The novel findings were as follows: (1) although the low to deficient serum 25[OH]D increased in most participants after a summer season, the mean serum 25[OH]D concentration was still below recommended values in 66% of the participants. This was independent of sex or the capacity to metabolize glucose. (2) No differences were found in 25[OH]D levels measured in the first year compared to the follow-up year. (3) No correlations were found between 25[OH]D and body composition variables in men.

### 4.1. Effects of Sun Exposure on Vitamin D

This study enrolled participants who lived in the Stockholm area of Sweden, which is located at 59°N latitude. Previous studies have shown that vitamin D status is lower in healthy women at 57°N compared with their counterparts at 51°N [19], while other studies have found that exposure to sun at higher latitudes in different populations is sufficient to increase and maintain vitamin D status to within the recommended range [20, 21]. In the present population, sun exposure during summer was insufficient to increase vitamin D to recommended values in 66% of the participants. The results were confirmed during the follow-up year. There are several possible explanations to the insufficient increase of 25[OH]D. To get the maximum benefit from sun exposure, it is recommended to stay outside for at least 15 minutes between 10.00 a.m. and 3.00 p.m., when the number of UVB photons is highest (UVB wavelengths 280–305 nm) [22]. If outdoor activity is performed early in the morning or late in the evening, the effects of sunlight on vitamin D status are negligible [1]. The subjects in our study could have avoided direct sun exposure at the beneficial hours due to increased temperature at that time. Further, use of protective clothing and sunblock [23] may also have reduced the effect of sun exposure, although there is no consensus in the literature regarding sunscreen and its effect on vitamin D status [24, 25]. However, when it comes to the risk of developing malign melanoma due to sun exposure, the official recommendations to avoid direct sun exposure between 10.00 a.m.–3.00 p.m. are very clear in the Nordic countries.

### 4.2. Associations between Body Fat and Vitamin D

An association between a high amount of body fat and low concentrations of 25[OH]D has been reported in many studies [26–28]. We found a weak inverse correlation between 25[OH]D and body composition variables in the whole study population. In multiple linear regression analyses, FM (kg) independently influenced 25[OH]D in the study population, consistent with results from a previous study [29]. However, gender-specific multiple linear regression analyses showed that FM (%) independently influenced 25[OH]D in women, but not in men, which is inconsistent compared to previous studies. Jorde et al. [29] found an inverse association between vitamin D and BMI, in both gender, which we failed to replicate in two consecutive summer seasons. It has also been shown that low vitamin D is associated with a higher prevalence of metabolic syndrome and T2DM [2, 28]. The risk seems to increase during winter, when vitamin D status decreases [30]. Our results confirmed that vitamin D status declines during winter seasons, as 84% of the participants had low to deficient 25[OH]D before summer. However, we did not detect any associations between glucose intolerance and 25[OH]D concentrations. We were only able to find weak to moderate correlations between body composition variables and 25[OH]D in women with NGT.

### 4.3. Vitamin D in Food and in Supplements

Dietary intake of fatty fish has played an important role in ensuring adequate vitamin D status in the Nordic countries [31]. In our study population, self-reported fatty fish intake was not correlated with serum 25[OH]D. In multiple linear regression analyses, supplementation with vitamin D independently influenced 25[OH]D in the whole study population and in women with NGT. However, only 21 participants reported taking supplements.

### 4.4. Clinical Considerations

There is mounting evidence that a high concentration of 25[OH]D is important for ensuring overall health and bone and cardiovascular health, as well as the immune system [32–34]. What remains a topic of debate is the classification of vitamin D deficiency. There are substantial inconsistencies regarding cutoff values for 25[OH]D “deficiency,” “optimal,” and “safe” values. Further, the assessment of vitamin D as 25[OH]D concentration is made using a variety of methods, which makes it difficult to compare [35]. United States Institute of Medicine has defined vitamin D deficiency as <30 nmol·L^−1^. However, such recommendations may be too conservative, according to the existing literature which suggests 25[OH]D concentrations above 120 nmol·L^−1^ for optimal health [36]. The recent literature seems to have reached a middle ground suggesting a range of 75–125 nmol·L^−1^ [37] for this outcome. Furthermore, there has been a growing focus on specific outcomes such as muscle function and the prevention of falls and fractures. Optimal 25[OH]D concentrations might be tissue-dependent and may not be the same for bone function and endocrine health [38]. In addition, several factors that influence vitamin D status must be taken into consideration. These factors include the gastrointestinal absorption of vitamin D, sex, amount of body fat, age, genetic heritage, diseases, and medications [9, 39].

### 4.5. Strengths and Limitations

The strength of this study is that the participants were homogenous and lived in the same area. In total, 68 of the participants underwent follow-up the subsequent year confirming findings from the first year. The limitations of this study include methods used to measure vitamin D intake via diet and supplementation. Further, there is a lack of detailed information about timing and outdoor activity as well as use of sunscreen products or protective clothing that could have limited the efficacy of UVB exposure.

## 5. Conclusions

The results of the present study demonstrated that, at 59°N latitude in Sweden, vitamin D levels were low in the spring and increased significantly during the summer, although not adequate according to recommended levels. Vitamin D was to some extent affected by body fat and supplementation, but not age, sex, or glucose metabolism status. This information is of clinical relevance since it has been assumed that sun exposure during the summer in Nordic countries is sufficient to reach recommended concentrations of 25[OH]D. Based on our results, efficient and appropriate vitamin D supplementation-protocols during both summer and winter should be discussed to improve vitamin D status in people living at higher latitudes and prevent decreased vitamin D values during the winter season.

## Figures and Tables

**Table 1:** Vitamin D (nmol/L) before and after summer in men and women classified by glucose tolerance status.

Vitamin D (nmol/L)	Before summer mean (SD)	After summer mean (SD)	*P* value
Men NGT *n*=40	53.3 ± 19.0	64.0 ± 22.0	<0.001
Men IGT + T2DM *n*=31	54.9 ± 27.4	69.2 ± 27.0	<0.001
Women NGT *n*=62	56.5 ± 17.8	67.9 ± 17.7	<0.001
Women IGT + T2DM *n*=25	55.0 ± 27.3	62.4 ± 19.4	0.004
All participants *N*=158	55.1 ± 21.7	66.3 ± 21.0	<0.001
Second summer season *N*=68	56.0 ± 22.0	67.5 ± 22.2	<0.001

Values are reported as mean ± standard deviation. Abbreviations: NGT, normal glucose tolerance; IGT, impaired glucose tolerance, T2DM, type 2 diabetes mellitus.

**Table 2:** Measures of glucose metabolism and body composition at baseline and their relationship (Pearson correlation coefficients) with vitamin D at baseline and its seasonal change during summer.

Variable	Mean values (SD)	Vitamin D before summer	Seasonal change in vitamin D
		*r*(*P*)	*r*(*P*)
OGTT (mmol/L)	5.86 (1.08)	0.04 (*P*=0.610)	0.02 (*P*=0.790)
OGTT 2 h mean (mmol/L)	9.36 (3.76)	–0.01 (*P*=0.905)	0.02 (*P*=0.838)
Insulin^‡^	56.29 (38.09)	–0.07 (*P*=0.371)	–0.03 (*P*=0.683)
HbA1c (mmol/L)	5.00 (0.73)	–0.00 (*P*=0.990)	–0.00 (*P*=0.993)
Fat mass (%)^†^	39.51 (8.15)	–0.10 (*P*=0.204)	–0.15(*P*=0.064)
Fat mass (kg)^†^	33.32 (8.71)	–0.21 (*P*=0.011)	–0.08 (*P*=0.326)
Fat mass index (kg·m^−2^)^†^	11.46 (3.36)	–0.18 (*P*=0.026)	–0.08 (*P*=0.337)
Fat-free mass (kg)^†^	51.04 (10.11)	0.07 (*P*=0.369)	0.14 (*P*=0.085)
Fat-free mass index (kg·m^−2^)^†^	17.18 (20.03)	0.10 (*P*=0.207)	0.21 (*P*=0.009)
Weight (kg)	84.36 (12.03)	–0.21 (*P*=0.009)	0.06 (*P*=0.467)
Waist (cm)^†^	97.42 (10.60)	–0.17 (*P*=0.043)	–0.02 (*P*=0.819)
Hip (cm)^†^	104.42 (7.37)	–0.24 (*P*=0.003)	–0.07 (*P*=0.382)
BMI (kg·m^−2^)	28.87 (3.61)	–0.20 (*P*=0.010)	0.03 (*P*=0.708)
Age (y)	60.30 (4.88)	0.08 (*P*=0.294)	–0.09 (*P*=0.226)
Height (cm)	171.10 (9.08)	–0.05 (*P*=0.548)	
Fish intake	1.90 (0.72)	0.12 (*P*=0.127)	
Supplementation of vitamin D	64.20 (20.11)	0.16 (*P*=0.039)	–0.11 (*P*=0.146)

Abbreviations: HbA1c, glycated haemoglobin; OGTT, oral glucose tolerance test. *N* = 158 if not otherwise stated; ^†^*N*=150, ^‡^*N*=155.

**Table 3:** Determinants of vitamin D (nmol/l) identified by multivariate regression analysis in 150 subjects. The overall *R*^2^ for the model was 0.13.

Variable	RC	*P* value
Fat (kg)	−0.27	0.003
Vitamin D supplementation	0.20	0.013
Age	0.61	0.460
Sex	−0.14	0.117
Glucose metabolism NGT/IGT/T2DM	0.09	0.288
Fish intake per week	0.13	0.115

*R* ^2^: explanatory value of the model; RC: standardized regression coefficient.

**Table 4:** Determinants of D vitamin D (nmol/l) identified by multivariate regression analysis in women (*n*=82) and men (*n*=68). The overall *R*^2^ for the model (women = 0.17) (men = 0.12).

Variable	Women		Men	
	RC	*P* value	RC	*P* value
Fat (%)	–0.28	0.012	–0.19	0.109
Vitamin D supplementation	0.23	0.030	0.19	0.126
Age	0.16	0.136	–0.09	0.448
Glucose metabolism NGT/IGT/T2DM	0.05	0.633	0.11	0.354
Fish intake per week	0.04	0.705	0.23	0.070

*R* ^2^: explanatory value of the model; RC: standardized regression coefficient.

## Data Availability

The data used to support the findings of this study are included within the article.

## Abbreviations

BMI: Body mass index
WT: Body weight
FM: Fat mass
25[OH]D: 25-Hydroxyvitamin D
IGT: Impaired glucose tolerance
NGT: Normal glucose tolerance
OGTT: Oral glucose tolerance test
SD: Standard deviation
T2DM: Type 2 diabetes mellitus
UVB: Ultraviolet B.
